# Body Composition Assessment in Pediatric Cystic Fibrosis in the Highly Effective Modulator Therapy Era—A Pilot Study

**DOI:** 10.3390/nu18152557

**Published:** 2026-08-05

**Authors:** Michelle Yavelow, Athanasios Tsalatsanis, Marisa Couluris, Danielle Reynolds, Racha T. Khalaf

**Affiliations:** 1 Division of Pediatric Gastroenterology, Hepatology and Nutrition, Department of Pediatrics, University of South Florida, Tampa, FL 33612, USA; khalafr@usf.edu; 2 Division of Pediatric Pulmonology, Department of Pediatrics, University of South Florida, Tampa, FL 33620, USA; 3 Office of Research, Research Methodology and Biostatistics Core, University of South Florida, Tampa, FL 33612, USA; atsalats@usf.edu; 4 Division of Pulmonology, Department of Pediatrics, University of South Florida, Tampa, FL 33612, USA; mcouluri@usf.edu; 5 USF Diabetes and Endocrinology Center, Health Informatics Institute, University of South Florida, Tampa, FL 33612, USA; dgomez1@usf.edu

**Keywords:** cystic fibrosis, body composition, lung function, pediatrics, fat mass, bioelectrical impedance analysis, dual-energy X-ray absorptiometry, modulator therapy, body mass index

## Abstract

**Background/Objectives:** The prevalence of obesity in cystic fibrosis (CF) has increased following the introduction of highly effective modulator therapy (HEMT). However, the impact of increased adiposity on the lung function of pediatric patients with CF (ppwCF) in the era of HEMT remains incompletely understood. Furthermore, while the body mass index (BMI) is the most commonly used nutritional marker in CF care, it has important limitations and may not accurately reflect body composition. **Methods:** In this cross-sectional pilot study, ppwCF aged 5–18 years completed Bioelectrical Impedance Analysis (BIA) while a subset completed a dual-energy X-ray absorptiometry (DXA) scan and spirometry. Normality was assessed using Shapiro–Wilk tests. Correlations, categorical association, agreement tests, Firth penalized logistic regression, and exploratory linear regression of continuous FEV1% predicted were used to examine relationships among anthropometric measurements, body compositions, and pulmonary function. **Results:** Thirty-four individuals completed study procedures (19 female (56%); mean age 9.85 (SD 3.58)). BIA and DXA-derived body fat percentages were strongly correlated, and categorical agreement was moderate (Cohen’s Kappa 0.574; *p*-value 0.005). In age and sex-adjusted linear regression, BIA fat percentage (β = −0.23; 95% CI, −1.09 to 0.64; *p* = 0.595) and DXA fat percentage (β = −0.47; 95% CI, −2.14 to 1.21; *p* = 0.552) were not significantly associated with FEV1% predicted. Similarly, Firth logistic models showed no statistically significant association between the adiposity category and reduced lung function. **Conclusions:** BIA-derived body fat measurements correlate with DXA-derived measurements in ppwCF and demonstrate feasibility for use in routine CF clinic visits. This pilot cohort did not show a statistically significant relationship between higher adiposity and poorer pulmonary function. However, the wide confidence intervals indicate substantial uncertainty. Larger prospective studies are needed to evaluate longitudinal relationships between body composition and lung function in the HEMT era.

## 1. Introduction

The advent of cystic fibrosis transmembrane conductance regulator (CFTR) modulator therapy has transformed the care of people with cystic fibrosis (pwCF), resulting in substantial improvements in nutritional status, pulmonary outcomes, and survival. Historically, malnutrition and impaired linear growth have been associated with poorer lung function in (pwCF, and nutritional interventions have focused on achieving and maintaining adequate weight gain [1,2]. Limited data from the pre-highly effective CFTR modulators therapy (HEMT) era suggest that pwCF who have overweight or obesity have an increase in forced expiratory volume in one second percent predicted (FEV1%pred) [3,4].

BMI has long served as the primary nutritional assessment tool in pediatric patients with CF (ppwCF), with BMI at or above the 50th percentile set as the target because of its positive correlation with lung function [1]. Since the advent of HEMT in 2019, health outcomes have improved, including the proportion of pwCF with a BMI above traditional targets. The relationship between elevated BMI and lung function is now less clear and is of clinical interest. Pediatric patients without CF and with obesity are at a higher risk of early onset diabetes, cardiovascular disease, and decreased lung function compared to children of a healthy weight [4,5]. Emerging evidence in CF implies that a higher BMI may not confer respiratory benefit [3,6,7].

A major limitation of BMI is its inability to distinguish between lean and fat mass. As a result, BMI may not accurately reflect nutritional status or body composition [8,9]. Body composition assessment can be a more sensitive marker of nutrition status when compared to BMI alone. Previous studies have shown that excess adiposity in the general population is inversely associated with lung function [3]. Data on the body composition of ppwCF at or above the 50th percentile BMI is limited [10].

Bioelectrical Impedance Analysis (BIA) can be a practical alternative to obtain information on lean mass and fat-free mass. Technological advances have improved the accuracy and accessibility of BIA, allowing for rapid, noninvasive measurement of fat mass and fat-free mass in outpatient settings [11]. Recognizing its utility, in 2020, the Academy of Nutrition and Dietetics recognized BIA as a tool for adult and pediatric CF to trend individual body composition parameters over time [8].

Currently, the Cystic Fibrosis Foundation (CFF) recommends that ppwCF obtain a dual-energy X-ray absorptiometry (DXA) scan to monitor bone mineral density at least once prior to reaching 18 years of age or if they are >8 years old if they meet the following criteria: <90% Ideal Body Weight, FEV1 < 50% predicted, glucocorticoids of ≥5 mg/day for ≥90 days/year, delayed puberty, or a history of bone fracture [12,13]. While bone health is a key parameter to assess in pwCF, DXA scans can assess nutritional status using parameters such as lean and fat mass that may further inform nutritional assessment [14,15]. Given the changing nutritional profile of pwCF in the HEMT era, a better understanding of body composition and its association with pulmonary outcomes can guide individualized nutrition interventions beyond the prior goals of high-calorie/high-fat diets.

The objective of this study was to assess body fat percentage in ppwCF using BIA and DXA-derived measurements and to investigate adiposity correlations with lung function measured with FEV1%pred in ppwCF.

## 2. Materials and Methods

### 2.1. Study Design and Participants

This cross-sectional study was conducted at the University of South Florida following Institutional Review Board approval. Written informed consent and assent were obtained from participants and/or their guardians as appropriate. Inclusion criteria consisted of pediatric patients 5–18 years of age with a confirmed diagnosis of CF via a chloride sweat test and/or CFTR genetic testing. Patients outside the specified age range, those without CF, and those with known concerns for disordered eating behaviors were excluded. A convenience sample was utilized limited by the clinic census of patients meeting inclusion criteria.

Demographic and clinical data were abstracted from the electronic medical record (EMR), including age, sex, CFTR genotype and mutation class, pancreatic insufficiency status, HEMT use, anthropometric data including BMI, mid–upper arm circumference (MUAC), body composition results including BIA and DXA results, post-BIA and post-DXA survey responses, and spirometry results (FEV1%pred, forced vital capacity (FVC), and forced expiratory flow at 25–75% of FVC (FEF25-75)). CFTR mutations were reported as class categories (I, II, III, IV, or V) [16]. Lung function was defined as a binary variable (normal (FEV1%predicted ≥ 80) versus obstructive (FEV1% predicted ≤ 79) [17,18,19].

Artificial intelligence was not utilized in the completion of the research or this manuscript.

### 2.2. Study Assessments

All procedures conducted were part of the standard of care except for the completion of BIA and patient experience survey. Weight and length measurements were obtained by clinic nursing staff using a Health O Meter professional scale and Veeder-Root and Holtain Limited stadiometers, respectively. BMI was categorized based on z-scores: mild malnutrition (−1 to −1.99), normal (−0.99 to 1.00), overweight (1.01–1.60), obese (>1.60). MUAC was obtained using measuring tape by a trained registered dietitian.

Body composition was assessed using Bioelectrical Impedance Analysis (BIA: Tanita DC-430U, Version DC4307661(0)1411FA, Japan) and, when available, dual-energy X-ray absorptiometry (DXA; Hologic; Model Horizon A (S/N 201098) USA). BIA was performed by the registered dietitian and provided estimates of body fat percentage and fat-free mass. The BIA device was readily available for use in clinics and validated for use in pediatric patients from 5 to 18 years of age. Per device manual instructions, BIA was not completed after any strenuous exercise, after excessive food or fluid intake, or when dehydrated. DXA scan completion was set for ppwCF aged 10–18 years that had yet to complete a baseline DXA scan. DXA scan was completed on fasted individuals (minimum of 4 h fasted). DXA was interpreted by a pediatric endocrinologist. BIA and DXA fat mass percentages were categorized using age and sex-scaled ranges defining underfat, normal, overfat, and obese provided in the manual of the BIA scale (Supplemental Materials—Figure S1) [20,21,22].

After BIA and DXA scan completion, patient experience surveys were administered (Appendix A—Table A1 and Table A2).

Pulmonary function testing was performed by a respiratory therapist using the Koko portable spirometer with spirometry software following the American Thoracic Society and European Respiratory Society technical standards [17,18]. Spirometry results were interpreted by a pediatric pulmonologist. Lung function was categorized as normal (FEV1% predicted ≥ 80%) or reduced (FEV1% predicted < 80%). Reduced lung function was further classified as mild (60–79%), moderate (40–59%), or severe (<40%).

### 2.3. Statistical Analysis

Participant characteristics and study measures were summarized descriptively. Continuous variables were reported using mean and standard deviation (SD) and median with min/max values. Categorical variables were reported using frequencies and percentages. Normality was assessed using the Shapiro–Wilk test before Pearson correlation analyses, which were used to evaluate the relationship between BMI and BIA-derived fat percentage in the full cohort and between BMI and DXA-derived fat percentage in the paired DXA subgroup. When a variable showed evidence of non-normality, Spearman rank correlation was also performed as a sensitivity analysis. Scatterplots were reviewed for linearity and influential observations. Agreement between continuous BIA and DXA measurements was evaluated using Bland–Altman analysis. Categorical associations were assessed using Fisher’s exact test, and agreement was summarized using Cohen’s weighted kappa.

Associations between body fat category and pulmonary status were examined using categorical and continuous outcome analyses. Lung function was categorized as normal (FEV1% predicted ≥ 80%) or reduced/obstructive (FEV1% predicted < 80%). Firth penalized logistic regression was used to estimate odds ratios and 95% confidence intervals for lung-function status, adjusted for sex. Exploratory linear regression models evaluated continuous FEV1% predicted in relation to BIA or DXA fat percentage. The analysis was conducted first unadjusted and then adjusted for age and sex. MUAC was compared with BIA and DXA using Spearman correlations for continuous measurements and Fisher’s exact test and weighted kappa after harmonizing categories as low, normal, or high. All analyses used available cases, were two-sided, and considered *p* < 0.05 as the statistical significance threshold. Given the pilot design, the results were considered exploratory and interpreted using effect estimates and 95% confidence intervals rather than *p*-values alone. No post hoc power calculation was performed; confidence interval width was used to communicate precision.

## 3. Results

### 3.1. Study Cohort Characteristics

A convenience sample of 34 individuals with CF who met the inclusion criteria were enrolled in this study. Participants had a mean age of 9.85 years (SD 3.58) and 19 (53%) were female. Most participants had Class II mutations (*n* = 16, 47.1%), were prescribed HEMT (*n* = 30, 88.2%), and had exocrine pancreatic insufficiency requiring enzyme replacement therapy (*n* = 26, 76.5%) (Table 1). Demographic data showed that 26 patients (76.5%) identified as White and not Hispanic or Latino, and 24 participants (70.6%) had public insurance. Fifteen were over 10 years of age and completed a DXA scan in addition to BIA.

Using the current guidelines for CF nutrition assessment, 62% (*n* = 21) had a BMI-for-age above the 50th percentile, with a mean z-score of 0.440 (SD 1.02). Similarly, 70% (*n* = 24) had a MUAC within the normal range.

Most participants (*n* = 21/32, 65.6%) had normal lung function, with FEV1%pred ≥ 80%. Mild reduction in lung function was present in eight participants (23.5%), while three met the criteria for moderate obstruction (8.8%).

### 3.2. Fat Percent Correlation Between DXA and BIA

BMI was not normally distributed (Shapiro–Wilk W = 0.865; *p* = 0.001), and BIA-derived body fat percentage showed no evidence of non-normality (W = 0.969; *p* = 0.436). BMI and BIA-derived body fat percentages were strongly positively correlated (Pearson analysis: r = 0.836; 95% CI: 0.694–0.915; *p* < 0.001; Spearman analysis: ρ = 0.783; *p* < 0.001).

**Figure 1:**
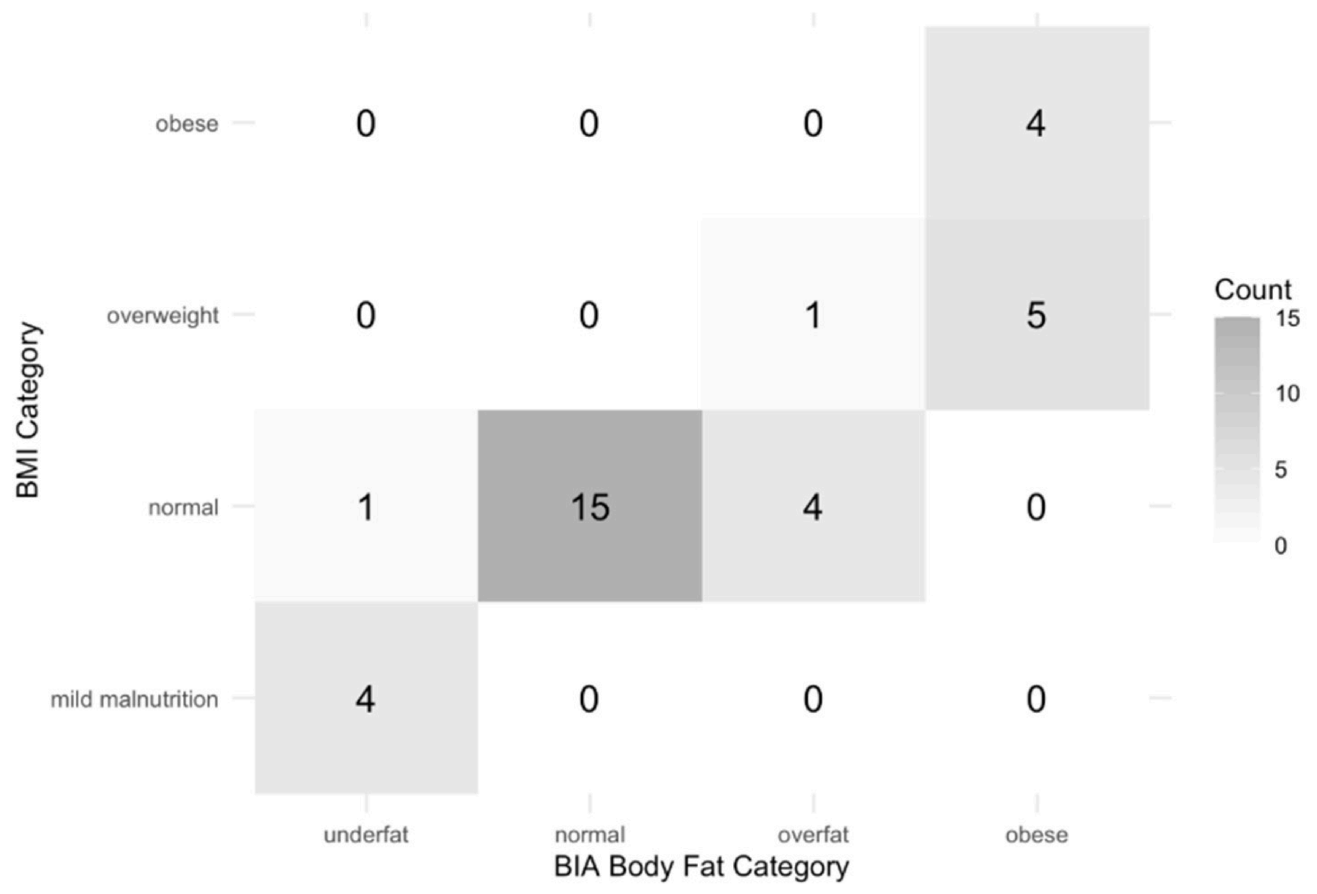
Body mass index (BMI) vs. Bioelectrical Impedance Analysis (BIA) classification.

Despite this strong association, substantial variability in body fat percentage was observed across similar BMI values, suggesting that BMI may not accurately reflect body composition. Agreement between BMI classification (mild malnutrition, normal, overweight, obese) and BIA-derived body fat classification was substantial (Cohen’s κ = 0.847; Figure 1).

**Figure 2:**
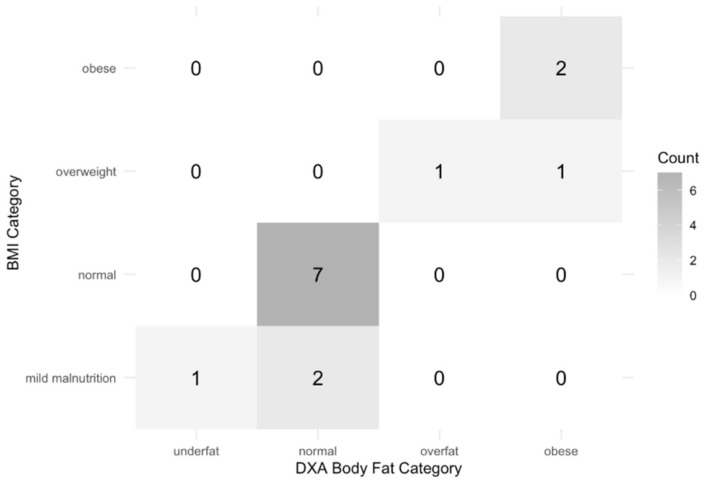
Body mass index (BMI) vs. dual-energy X-ray absorptiometry (DXA) classification.

Among participants who underwent a DXA scan (*n* = 15), neither BMI nor DXA-derived body fat percentages showed evidence of non-normality (Shapiro–Wilk *p* = 0.335 and *p* = 0.709, respectively). BMI (kg/m^2^) demonstrated a strong positive correlation with DXA-derived body fat percentage (Pearson r = 0.772; 95% CI 0.426–0.920; *p* < 0.001). There was a significant association with a moderate to substantial agreement when comparing BMI classification with DXA-derived body fat categorization (Fisher’s exact *p* = 0.002; Figure 2).

**Figure 3:**
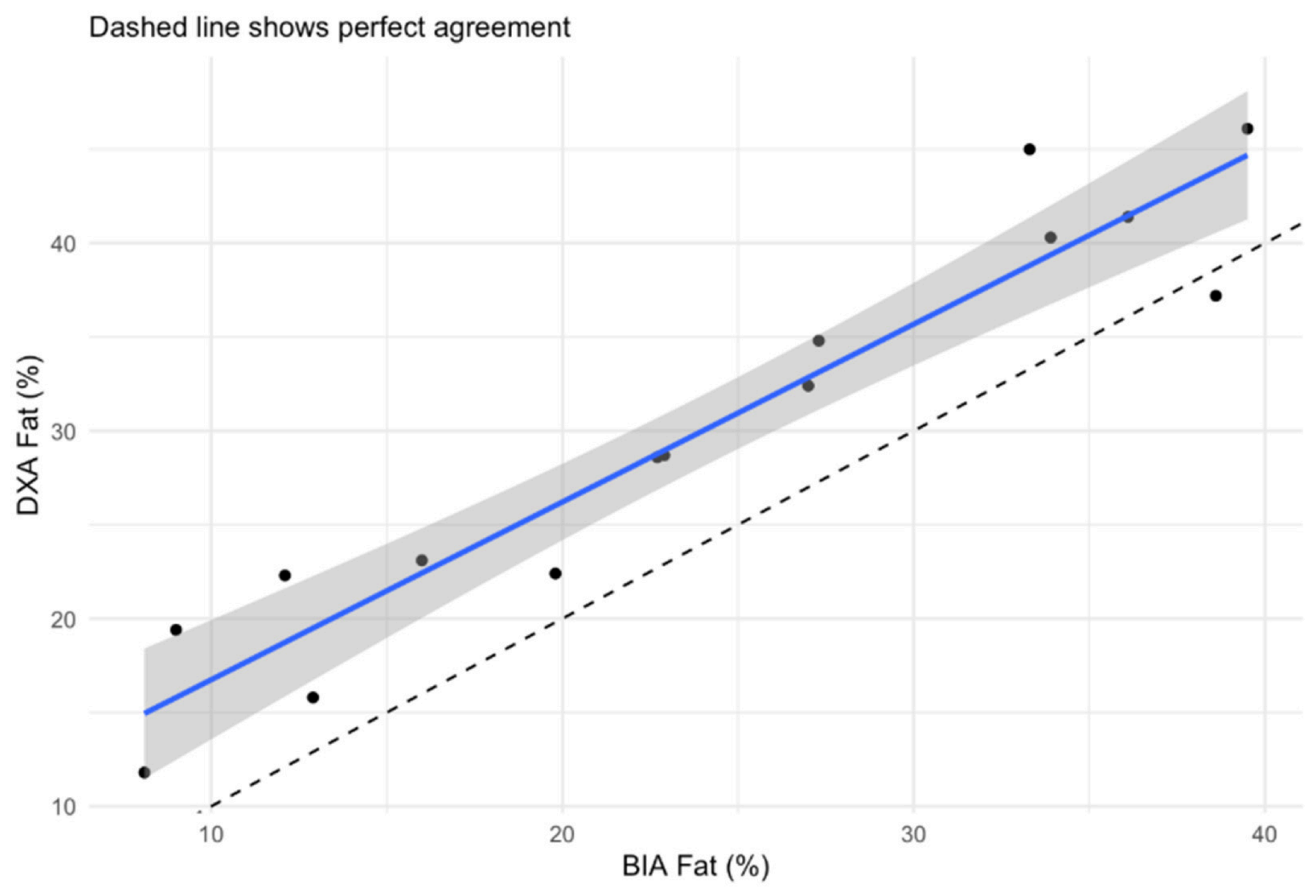
Bioelectrical Impedance Analysis (BIA) vs. dual-energy X-ray absorptiometry (DXA) for body fat percentage. The black dots represent each participant’s BIA against DXA fat percentage. The solid blue line is the fitted linear regression line showing the average relationship between BIA and DXA fat percentages. The shaded area is the 95% confidence interval for the estimated mean regression line. The dashed line shows perfect agreement.

Among participants who completed both assessments, BIA-derived and DXA-derived body fat mass percentages demonstrated a strong positive correlation, indicating that higher body fat measurements obtained by one method were generally associated with higher measurements obtained by the other (Figure 3). However, there was poor agreement between the two methods, suggesting that BIA and DXA fat percentages should not be considered interchangeable as continuous measures.

**Figure 4:**
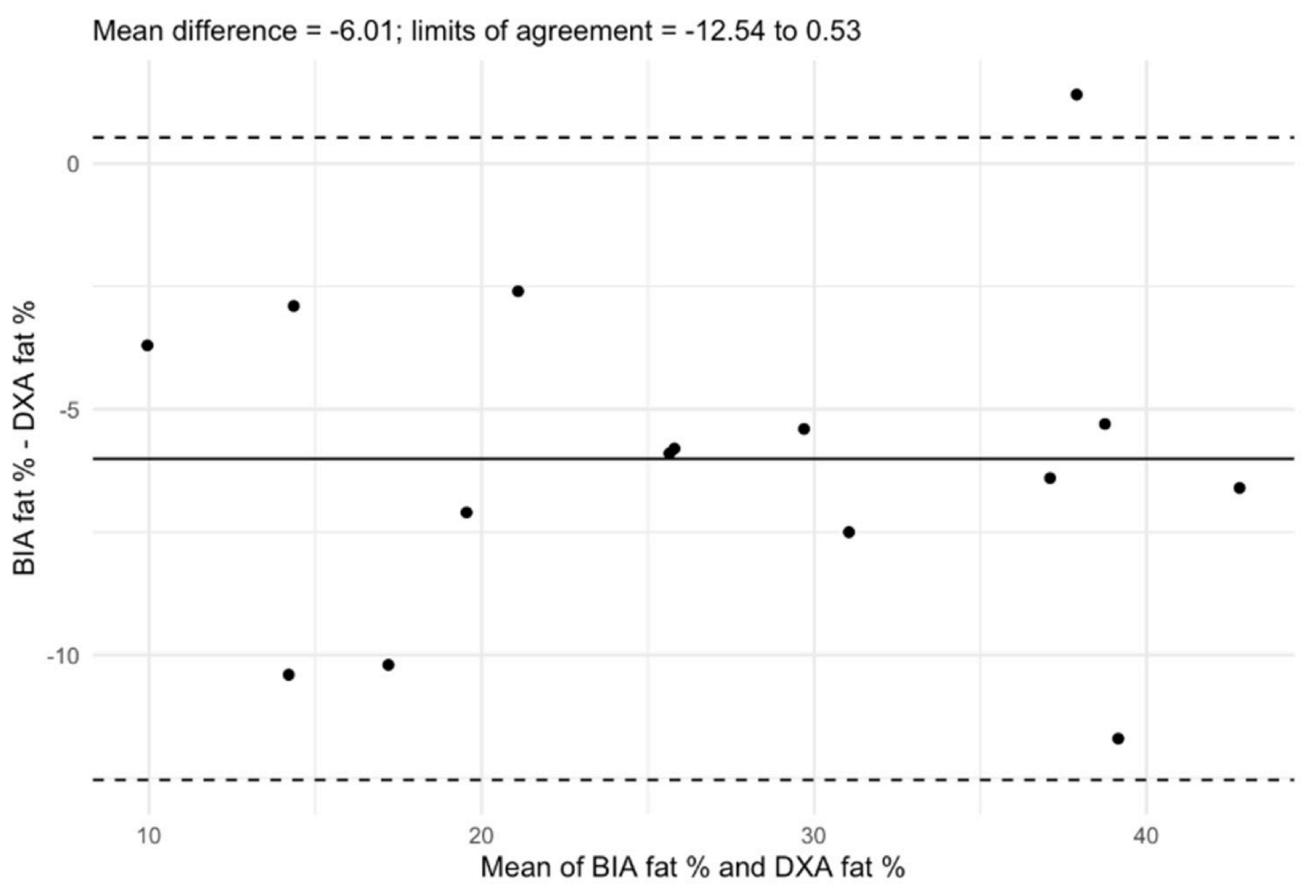
Bland-Altman plot: Bioelectrical Impedance Analysis (BIA) for fat percentage vs. dual-energy X-ray absorptiometry (DXA) for fat percentage. The black dots are individual patient data. The solid horizontal line represents the mean difference between BIA and DXA values. The dashed lines represent the 95% limits of agreement.

**Figure 5:**
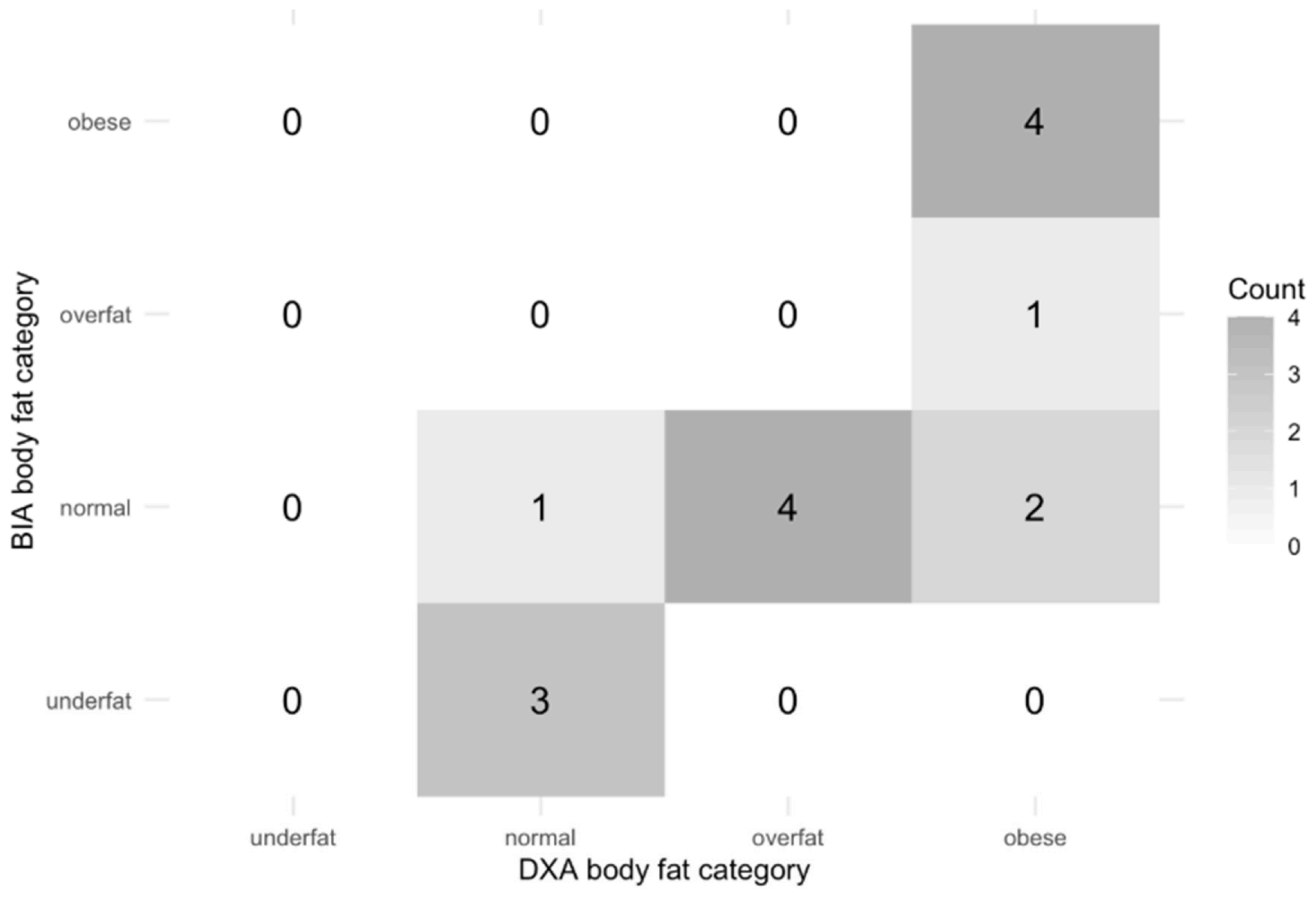
Bioelectrical Impedance Analysis (BIA) vs. dual-energy X-ray absorptiometry (DXA) for body fat classification.

When body fat percentage measurements were categorized as underfat, healthy, overfat, or obese, agreement between BIA and DXA classifications was moderate (Cohen’s κ = 0.574, *p* = 0.005) (Figure 4 and Figure 5). These findings suggest that while the two methods may differ in their absolute body fat estimates, they demonstrate reasonable concordance when used to classify individuals into clinically relevant adiposity categories.

### 3.3. Fat Mass Percent and Pulmonary Outcomes

Body fat classification derived from BIA and DXA was compared with lung function status as categorized by FEV1%pred values (FEV1%pred ≥ 80%) or obstructive (FEV1%pred < 80%). There was no statistically significant association between body adiposity classification (underweight, normal, overweight, obese) and lung function status for either body composition assessment method (Fisher’s exact *p* = 0.228 BIA; *p* = 1.000 DXA). The same pattern was observed in the sex-adjusted Firth logistic regression. In the BIA model, compared with the underfat group, the odds ratios were 2.91 (95% CI, 0.41–33.70; *p* = 0.293) for normal, 0.28 (95% CI, 0.00–6.49; *p* = 0.432) for overfat, and 1.87 (95% CI, 0.21–24.41; *p* = 0.580) for obese. In the DXA model, the corresponding odds ratios were 4.79 (95% CI, 0.17–845.14; *p* = 0.367), 14.98 (95% CI, 0.15–8005.22; *p* = 0.259), and 2.56 (95% CI, 0.05–534.65; *p* = 0.636), respectively.

**Figure 6:**
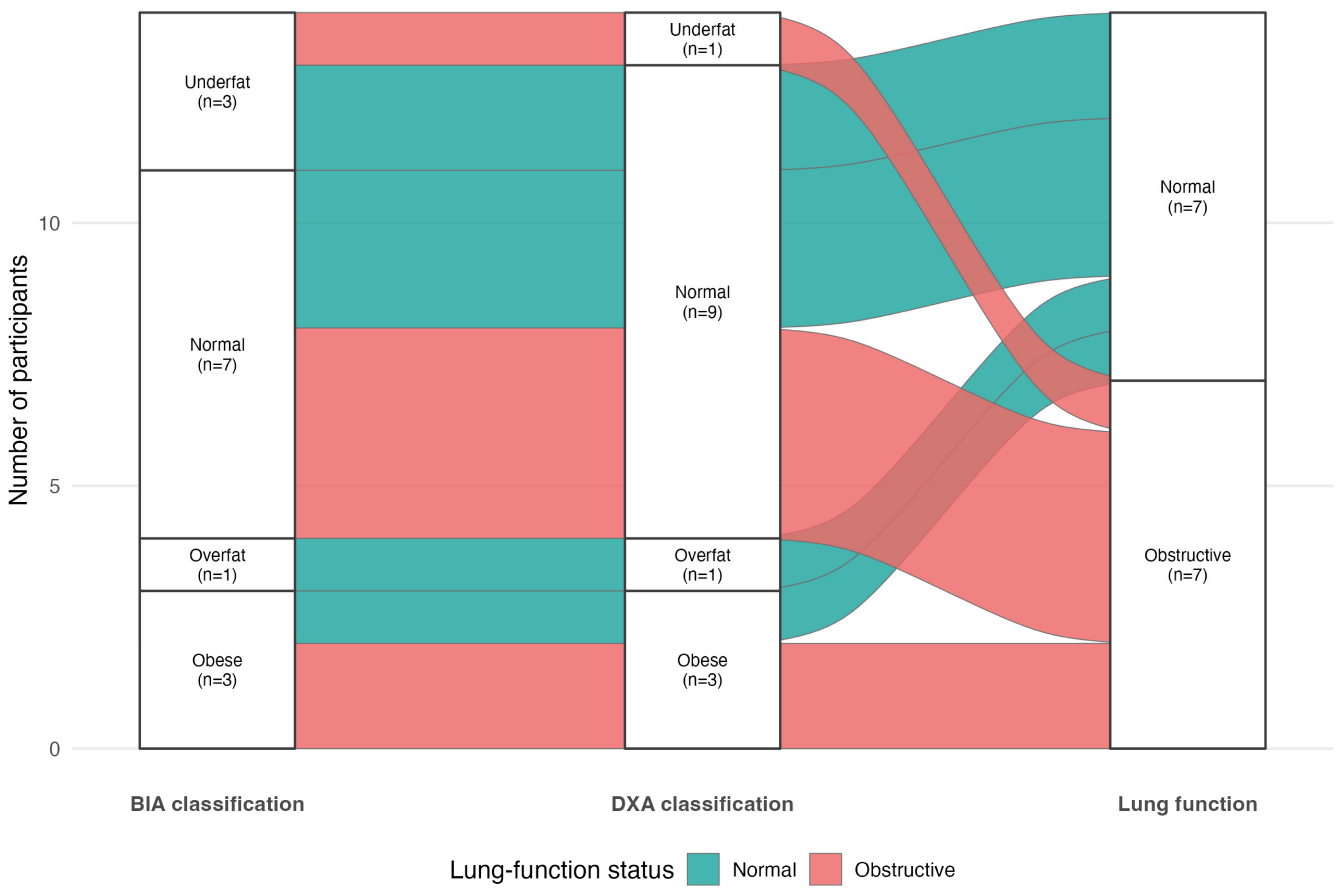
Sankey diagram. Flow of participants from Bioelectrical Impedance Analysis (BIA) to dual-energy X-ray absorptiometry (DXA) classification and lung function.

In the linear regression of continuous FEV1% predicted, the unadjusted BIA fat-percentage coefficient was −0.20 (95% CI, −1.00 to 0.60; *p* = 0.615), and the age and sex-adjusted coefficient was −0.23 (95% CI, −1.09 to 0.64; *p* = 0.595; *n* = 32). In the DXA subgroup, the unadjusted coefficient was −0.22 (95% CI, −1.56 to 1.13; *p* = 0.735), and the adjusted coefficient was −0.47 (95% CI, −2.14 to 1.21; *p* = 0.552; *n* = 15). While the analyses did not demonstrate a statistically significant relationship between fat percentage and lung function, modest associations in either direction cannot be excluded because of limited precision (Figure 6). Data reference for the Sankey diagram can be found in Appendix A (Table A3).

MUAC z-score was strongly correlated with BIA fat percentage (Spearman ρ = 0.824; *p* < 0.001) and DXA fat percentage (ρ = 0.879; *p* < 0.001). After harmonization into low, normal, and high categories, MUAC classification was associated with BIA classification (Fisher’s exact *p* < 0.001), whereas the MUAC–DXA categorical association was not statistically significant (*p* = 0.179). This comparison was limited by the 15-participant paired subgroup and sparse cells.

### 3.4. Participant Survey Data

Following completion of both BIA and DXA assessments, participants reported high acceptability of both procedures. Using a Likert scale from 1 to 5, with 1 being “strongly disagree” and 5 being “strongly agree”, most patients felt that the BIA assessment was quick (mean response 4.97, SD 0.17), easy to complete (mean response 4.91, SD 0.38), and painless (mean response 4.94, SD 0.34). All patients reported that the DXA scan was quick and easy to complete. The majority of patients expressed willingness to complete repeat testing in the future (91%).

Minor concerns related to BIA included discomfort associated with the removal of socks and the cold sensation of the scale on bare feet. There were no specific complaints from the patients after completing the DXA scan (Appendix A, Table A4 and Table A5).

## 4. Discussion

Since the advent of HEMT, pwCF have increased longevity that comes with improved lung function and increased growth velocity. There is a growing body of literature investigating alternative methods to assess health indicators in pwCF. Body composition is a parameter being investigated in pwCF that will trend over time, including the use of BIA and DXA [12]. As assessments are becoming more well defined in the adult population, there is a lack of knowledge on body composition in ppwCF and how this information correlates with the current primary health indicator, lung function.

This study found that BMI-for-age health targets were generally consistent with body fat classification measured with BIA, although there was notable inter-individual variability that remained. Neither categorical analyses, sex-adjusted Firth logistic regression, nor age and sex-adjusted linear regression demonstrated a statistically significant relationship between adiposity and pulmonary function. The very large confidence intervals, particularly for the 15-participant DXA subgroup, indicate substantial uncertainty and do not allow for reliable conclusions to be made about increased odds of reduced lung function. BIA and DXA measurements were strongly correlated but should not be considered interchangeable because agreement for continuous measurements was poor. These findings are exploratory and should be interpreted cautiously because of the small sample size and wide confidence intervals.

In 2019, Calella et al. found significant heterogeneity in the field of alternative assessments of anthropometrics in pwCF; however, the only methods with a sufficient body of literature are DXA and BIA [12]. Our center currently follows CFF guidelines and recommends DXA scans for bone density analysis [14]. Barriers to obtaining DXA scans include limited access to pediatric DXA scans, limited scheduling availability, off-site location, and additional missed school and workdays [23]. DXA reports vary and can typically only report on bone density and do not routinely include information on body composition. Merino et al. found that ppwCF on HEMT over time had increases to both body fat mass and fat-free mass associated with increased weight, measured with BIA [24]. This research can help expand standards of care to overcome these challenges to incorporate BIA and DXA beyond bone density scanning into annual screening for pwCF.

While the DXA scan is the gold standard of measuring and interpreting body composition, there is limited data available to interpret the body fat z-scores and percentiles generated from the DXA machine in the pediatric population. The DXA scan in this study was able to measure and quantify the percent lean mass in this population. The BIA scale utilized did not offer similar information and could not be directly compared. There is a growing body of evidence to suggest that lean mass is an important indicator of lung function in adults with CF [25,26,27]. Lean mass assessment and interpretation in the pediatric population have limited data to extrapolate healthy targets across the life stages. It is furthermore challenging to find consistent tools to use outside of the DXA scan to measure lean mass in the clinic setting, assessing beyond the two-component model of “fat mass” and “fat free mass” to differentiating fat-free mass to skeletal muscle mass, total body water, bone mass, and organ mass [28].

The strong correlations between MUAC z-score and both BIA and DXA-derived fat percentage suggest that MUAC may offer useful information where instrument-based body composition assessment is unavailable. The categorical MUAC–BIA association was statistically significant, whereas the MUAC–DXA association was not (interpretation of the MUAC–DXA association is limited by the small paired sample).

There is a growing body of research supporting the reliability and validity of DXA scans in the assessment of pediatric body composition. Specific challenges involving the pediatric population include variations in sexual maturation, height, and ethnicity. Efforts have been made to adjust for some of these differences; for example, calculators were used to adjust for height differences [29]. Using height-adjusted metrics to help improve the reliability of data would be recommended. In addition, accounting for Tanner stage and/or bone age may also help better understand and interpret the data as body composition changes significantly during growth and maturation. Despite limitations, DXA is still considered one of the most reliable techniques for measuring body composition in pediatric populations with overweight and obesity [30].

Throughout several decades of CF research, nutritional status and lung function have been closely monitored because both are critical determinants of survival. Before the introduction of HEMT, clinical management focused on preventing and treating malnutrition, with evidence supporting the maintenance of BMI at or above the 50th percentile to optimize lung function outcomes [31]. However, as the prevalence of overweight and obesity among pwCF has increased, emerging evidence suggests that there may be a threshold beyond which further increases in BMI no longer confer pulmonary benefits and may instead be associated with adverse effects on lung function [3,6,7]. Recent evidence suggests that ppwCF who are pancreatic-sufficient may have a detrimental effect on pulmonary function if their BMI is above the 85th percentile [32]. Similarly, data from the Canadian CF Registry demonstrated that although lung function improved with increasing weight, the magnitude of this benefit was substantially attenuated among individuals with obesity [7]. In this study, however, neither the categorical nor continuous-outcome analyses provided evidence that higher adiposity was associated with worse lung function. The logistic point estimates were unstable, non-monotonic across adiposity categories, and accompanied by very wide confidence intervals. The results should therefore be regarded as hypothesis-generating rather than evidence of increased odds.

These study results are limited by a small convenience sample; however, the recruitment was able to achieve a rate of 85% recruitment, with 13% (*n* = 5) declining to participate in the study and *n* = 1 not present in the clinic during the recruitment period. Per manufacturer guidelines, the BIA scale used in this study can assess body composition for pediatric patients aged 5–18 years. The pediatric CF center also found that with preparatory guidance and coaching, patients starting at age 5 can successfully complete the pulmonary function tests. The European CF Society recommends initial assessment starting at ages 8–10 [33]. This center utilizes the age of 10 for initial screening, and this was mirrored in the inclusion criteria for the DXA arm. A measurement of triceps skin fold could be used to calculate arm muscle area and arm fat area using a skinfold caliper. This device was not available for use in this study to further correlate with BIA; however, it could be used in future investigations.

Further, DXA scans were not completed on the same day as the BIA assessment, with an average time of 123 days between BIA completion and DXA scan completion. Time differences were complicated by limited time slots for endocrinology assessment at monthly clinics for patient-specific use of the DXA scan, delayed scheduling of DXA scan appointments, and clinic closure during one of the 12 potential clinic times during the study. Within this time frame, using Bland–Altman analysis, there was a mean difference of 2.2 kg in weight. Over this time, we also saw a change in the number of patients meeting overweight and obese criteria based on percent body fat percentage assessment (increase from *n* = 5 to *n* = 11). Given the expected growth and maturation in this pediatric cohort, these differences likely reflect physiological change over time rather than measurement discordance alone. All *n* = 3 patients that initially met underfat assessment at the time of their BIA did meet normal body fat percentage assessment at the later time of their DXA. Therefore, direct comparison of values is significantly limited, and future comparison studies should have the BIA and DXA completed on the same day.

## 5. Conclusions

In this study, BIA-derived body fat measurements were associated with DXA-based assessments and were feasible to obtain during routine CF clinic visits in pediatric patients at an age as young as 5 years. These findings support BIA as a practical noninvasive tool for body composition assessment in pediatric CF care and its potential utility for longitudinal monitoring across the pediatric age spectrum.

As CF care continues to evolve in the HEMT era, increasing longevity and rising rates of overweight and obesity highlight the need for more precise and accessible measures of body composition beyond BMI. This pilot study did not demonstrate a statistically significant association between body fat and lung function. Strong MUAC correlations with BIA and DXA support further study of MUAC as a complementary option where instrument-based testing is not available. Larger longitudinal studies incorporating more sensitive measures of early lung disease are needed to better define the relationship between body composition and pulmonary outcomes.

## Figures and Tables

**Table 1:** Descriptive statistics.

	Overall (*n* = 34)
**Age (years)**	
Mean (SD)	9.85 (3.58)
Median [Min, Max]	9.50 [5.00, 17.0]
**CF Mutation Class**	
1	1 (2.9%)
1, 2	9 (26.5%)
1, 4	1 (2.9%)
2	16 (47.1%)
2, 3	3 (8.8%)
2, 4	2 (5.9%)
4	1 (2.9%)
4, 5	1 (2.9%)
**Sex**	
Female	19 (55.9%)
Male	15 (44.1%)
**Insurance Type**	
Private	10 (29.4%)
Public insurance	24 (70.6%)
**Race**	
Asian	1 (2.9%)
Black or African American	3 (8.8%)
Cuban	3 (8.8%)
Other	1 (2.9%)
White	26 (76.5%)
**Ethnicity**	
Hispanic or Latino	8 (23.5%)
Not Hispanic or Latino	26 (76.5%)
**On Modulator Therapy**	
No	4 (11.8%)
Yes	30 (88.2%)
**Exocrine Pancreatic Insufficient**	
No	8 (23.5%)
Yes	26 (76.5%)

## Data Availability

The raw data supporting the conclusions of this article will be made available by the authors on request.

## Supplementary Materials

The following supporting information can be downloaded at: https://www.mdpi.com/article/10.3390/nu18152557/s1, Figure S1. Bioelectrical Impedance Analysis (BIA) reference for body fat percentage assessment.

## Abbreviations

The following abbreviations are used in this manuscript:

CFTR	Cystic Fibrosis Transmembrane Conductance Regulator
CF	Cystic Fibrosis
pwCF	Patients with Cystic Fibrosis
HEMT	Highly Effective Modulator Therapy
FEV1%pred	Forced Expiratory Volume Percent Predicted
BMI	Body Mass Index
ppwCF	Pediatric Patients with Cystic Fibrosis
CFF	Cystic Fibrosis Foundation
DXA	Dual-energy X-ray Absorptiometry
MUAC	Mid–Upper Arm Circumference
BIA	Bioelectrical Impedance Analysis
FVC	Forced Vital Capacity
FEF	Forced Expiratory Flow
SD	Standard Deviation
IOS	Impulse Oscillometry
LCI	Lung Clearance Index

## Appendix A

**Table A1:** Bioelectrical Impedance Analysis completion survey.

	Strongly Disagree	Disagree	Neutral	Agree	Strongly Agree
The BIA test was quick	1	2	3	4	5
The BIA test was easy to complete	1	2	3	4	5
The BIA test was painless	1	2	3	4	5
I would be happy to complete the BIA test again	YES		NO		

**Table A2:** Dual-energy X-ray absorptiometry.

	Strongly Disagree	Disagree	Neutral	Agree	Strongly Agree
The DXA test was quick	1	2	3	4	5
The DXA test was easy to complete	1	2	3	4	5
The DXA test was painless	1	2	3	4	5
I would be happy to complete the DXA test again	YES		NO		

**Table A3:** Participant pathways displayed in the BIA–DXA–lung-function Sankey diagram.

BIA Category	DXA Category	Lung Function	*n*	%
Underfat	Underfat	Obstructive	1	7.1
Underfat	Normal	Normal	2	14.3
Normal	Normal	Normal	3	21.4
Normal	Normal	Obstructive	4	28.6
Overfat	Overfat	Normal	1	7.1
Obese	Obese	Normal	1	7.1
Obese	Obese	Obstructive	2	14.3

**Table A4:** Bioelectrical Impedance Analysis (BIA) post-survey results.

	Overall
	(*n* = 34)
**The BIA test was quick.**	
Mean (SD)	4.97 (0.171)
Median [Min, Max]	5.00 [4.00, 5.00]
**The BIA test was easy to complete.**	
Mean (SD)	4.91 (0.379)
Median [Min, Max]	5.00 [3.00, 5.00]
**The BIA test was painless.**	
Mean (SD)	4.94 (0.343)
Median [Min, Max]	5.00 [3.00, 5.00]
**I would be happy to complete the BIA test again (1 = yes, 2 = no)**	
Mean (SD)	1.09 (0.288)
Median [Min, Max]	1.00 [1.00, 2.00]

**Table A5:** Dual-energy X-ray absorptiometry (DXA) post-survey results.

	Overall
	(*n* = 15)
**The DEXA test was quick.**	
Mean (SD)	5.00 (0)
Median [Min, Max]	5.00 [5.00, 5.00]
**The DEXA test was easy to complete.**	
Mean (SD)	5.00 (0)
Median [Min, Max]	5.00 [5.00, 5.00]
**The DEXA test was painless.**	
Mean (SD)	4.27 (0.799)
Median [Min, Max]	4.00 [3.00, 5.00]
**I would be happy to complete the DEXA test again (1 = yes, 2 = no)**	
Mean (SD)	1.00 (0)
Median [Min, Max]	1.00 [1.00, 1.00]
Missing	1 (6.7%)
